# Research Progress on Expansive Soil Cracks under Changing Environment

**DOI:** 10.1155/2014/816759

**Published:** 2014-06-11

**Authors:** Bei-xiao Shi, Cheng-feng Zheng, Jin-kun Wu

**Affiliations:** ^1^College of Resource, Hebei University of Engineering, Handan, Hebei 056038, China; ^2^Geotechnical Engineering Department, Nanjing Hydraulic Research Institute, Nanjing, Jiangsu 210024, China; ^3^College of Water Conservancy and Electric Power, Hebei University of Engineering, Handan, Hebei 056021, China

## Abstract

Engineering problems shunned previously rise to the surface gradually with the activities of reforming the natural world in depth, the problem of expansive soil crack under the changing environment becoming a control factor of expansive soil slope stability. The problem of expansive soil crack has gradually become a research hotspot, elaborates the occurrence and development of cracks from the basic properties of expansive soil, and points out the role of controlling the crack of expansive soil strength. We summarize the existing research methods and results of expansive soil crack characteristics. Improving crack measurement and calculation method and researching the crack depth measurement, statistical analysis method, crack depth and surface feature relationship will be the future direction.

## 1. Introduction

Expansive soils are composed primarily of hydrophilic clay minerals, such as Montmorillonite, and with significant swelling and shrinking characteristics. Compared with the common clay, expansive soil has three characteristics, expansive, crack and over-consolidation. Crack is concentrated expression of expansion and overconsolidation. With the decline of water content expansive soil will shrink and result in crack; the changing of environment leads to drying and wetting effect and the crack will further develop. The release of expansive soil overconsolidated stress will also lead to crack propagation. The crack destroys the integrity of expansive soil, causes seepage, and results in soil swelling and crack aggravation. Therefore, crack is the key factor of the stability of expansive soil slope. Expansive soil is unsaturated soil with the special engineering properties, there is nearly hundred years of history study about the expansive soil basic physical and mechanical properties, engineering application, and the administer method form the early 20th century. Unsaturated expansive soil basic theory has matured gradually; a lot of extraordinary models were used in project successfully. However, the crack plays a key role on the expansive soil intensity, deformation, and seepage; however, the study about it has been slow. Therefore, it is necessary to summarize the research production, illustrate the study blind spot and difficulty, and point out the research work in future.

## 2. Basic Characteristics of Expansive Soils

Expansive soil is a special soil with swelling shrinkage characteristics, overconsolidation properties, and crack characteristics; swelling shrinkage characteristics are mostly source of expansive soil engineering disease. For expansive soil study, many scholars [1–4] through many experiments that studied the swelling and shrinking mechanism and the influence rule of expansive soil from the drying and wetting cycles and swelling shrinkage anisotropic, proved the swelling and shrinking deformation to be irreversible, and putting that forward explained the swelling shrinkage essence of the expansive soil using the seepage press theory in physics, chemistry, and suction potential. Gong et al. [4] and Xie et al. [5] point out that swelling shrinkage reduced the expansive power. People reach a consensus that the factor of initial dry density, water content, and pressure caused swelling shrinkage influences to be regular [6]. Deposited expansive soil come into being overconsolidation soil; because of the upper soil erosion in the history, the soil has small natural void ratio, big degree of density, and initial structure strength characteristics. Expansive soil crack develops because of unloading effect; high initial strength and low seepage character are the external features about natural expansive soil overconsolidation. The relation of crack, expansion and overconsolidation is closed; crack destroys the integrality of soil, brings seepage aggravating the expansive soil welling shrinkage and expands crack more.

## 3. Necessity of Expansive Soil Cracks Study

### 3.1. Crack Produce

For natural expansive soil, damage at the process of soil's generation gives rise to soil natural crack; changing environment come into being dry-wet cycles, and the dry-wet cycle occurs on the soil in a certain depth, soil undergoes swelling shrinkage frequently and natural crack appears or expands gradually. With the water evaporation continuing, soil shrinkage measurement amplifying continues, the secondary crack forming little by little. Expansive soil's high shrinkage and low seepage character are the main cause; unit of soil surface layer shrinks first when soil evaporates, vertical direction soil shrinks freely because is no restriction, but the horizontal direction soil shrinkage is restricted by the interacting soil inside. When the upper soil water loss for evaporating the water content at below soil has no marked change, as a result, the upper and below soil shrinkage is inhomogeneous and the soil crack appears. The inhomogeneous shrinkage many times lead to the crack of expansive soil produced.

### 3.2. Crack Growth Progress

The transformation will emerge when vertical crack formed because of external condition changing.

**Figure 1:**
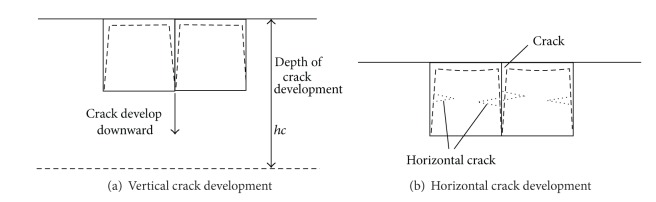
Producing and developing of fissuring of expansive soil.

1. Vertical crack surface formed; the touching area with the external air is amplified; horizontal shrink aggravate obviously and the vertical shrink continuing, crack will continue to expand downward until some balance is attained.  Figure 1(a) shows the process of crack developing.
2. Soil shrinkage aggravate inside horizontal cracking, but soil shrinkage is slowly near the vertical crack surface and far away from the vertical crack surface, inhomogeneous shrinkage at the vertical crack will lead to new horizontal crack shaping.  Figure 1(b) shows that.
3. Expansive soil exposes atmosphere influenced by the weather changes. Expansive soil will be loose with the precipitation infiltration to the soil, absorb water, and saturate soil gradually. Evaporation continues for a long time, the surface water and the free water of layer soil grow vapors into atmosphere, and the status of soil turns saturate into unsaturate. Natural expansive soil seeps low, and the layer soil will produce shrinkage crack with the evaporation continuing; yet the deep soil is not affected. Rain dips into soil along the crack; if rain falls once more after the crack is produced, the crack makes the deeper expansive soil swelling easy and provide the pass for water evaporating. Moreover, under the capillarity of crack, the water in soaking expansive soil rises repeatedly; the crack grows deeper step by step until attaining a balance. That is to say, the change of fine and rain promotes the crack emerging and growth.

## 4. Cracks on the Strength of the Control Action

Terzaghi [7] paid attention to the crack growth effect on soil strength initially and pointed out that the crack is the structure character of overconsolidation. Skenpton [8] found out that crack would cause stress concentration and the soil would be destroyed after stress exceeds the peak value of shear strength. Yao et al. [9], Bao [10], and Yin et al. [11] studied the crack effect on strength, deformation, and seepage; they consider that the strength of expansive soil exists; not only size effect but also alteration and reduction characteristic are restricted by crack. A lot of studies indicated that existing crack destroyed the soil's integrity, declined the strength of soil, and weakened the sliding ability; on the other hand, the crack provides the favorable pass for the water seepage and evaporation. The water pours into crack and provides penetrability when it is raining, the water pressure will be magnified if seepage in adverse slope. These two points reduce the safety factor and bring the notable influence to the stability of the slope. The swelling of expansive soil leads to the crack production and development; releasing the strength of expansive soil overconsolidation during excavation will cause crack development; changing of water content gives rise to expansive soil deformation and cracking. All of these can explain that the crack characteristic is reflect on swelling and over-consolidation, the crack is the key factor of the expansive soil slope stability and controls the strength of expansive soil.

## 5. Study on Expansive Soil Cracks at Present

### 5.1. Measure of Crack Observation

Crack exists in the soil interior; the change of outside condition leads the crack to open or close; the process of change is complex and direct or indirect observation is difficult. At present, describing and analyzing the process of the crack developing cannot be accurate; with the study of crack, achievements about the crack of statistical analysis, morphological description and the evolvement are regularly satisfactory. Lu et al. [12] studied the expansive soil evolvement regularly under drying and wetting cycles using CT; they put forward crack damage variable based on the number of CT and the rule of volume change accumulative total. Yi et al. [13] studied the fractal characteristic of expansive soil using fractal theory; they consider that the fractal number related to the strength of expansive soil. Yuan et al. [14] presented the simplified crack model; they observed expansive soil using optical microscopy and discussed the feasibility of quantifying the crack for gray entropy. Ma et al. [15] researched the cracks evolution using the humidity test device and analyzed the test conclusion. Chen et al. [16] studied the deformability and stability of expansive soil slope through centrifugal model test, analyzed the process of crack occurrence and development, and discussed the infiltration effect on expansive soil slope stability. Tang and Kong [17] morphologically analyzed and quantified the process of cohesive crack formation. Gong et al. [18] used the resistivity method to study development of expansive soil crack.

From all the above, we observe the crack directly through taking a picture; operation is simple but human factors is very complex. Human factor is less than the others using the resistivity method,but it is strongly affected by soil characteristic, such as void ratio or water content. Measuring the dynamic crack, quantitative and lossless through the CT and ultrasonic, the accuracy is higher but the instrument is expensive and operation requirement is higher than the average. In addition, the achievement of study is restricted to laboratory test; effective obvious method of crack at spot is absent. Cracks quantification, study of cracks expansion and the evolution of strength decay is blank especially under the changing environment, study of the crack expansion and the evolution of strength decay is blank.

### 5.2. Cracking Count Calculate

For expansive soil engineering applied research, people start from the analysis of slope stability at first. Any engineering examples of expansive soil slope instability, regardless if surface cracks can be seen or not (be covered or crack too small), proved that instability is the result of cracking [19]. So,the expansive soil slope stability be decided by soil crack and the level of cracking. The crack depth at longitudinal direction is not accurate in practical engineering; the expansive soil crack depth can be 4 meters. No matter what by replacing or adding cement-based materials to expansive soil, or other methods, the depth of expansive soil slope destroyed must be clear. Otherwise, enormous economic waste and huge economic burden will be brought out; effect of treatment cannot be sure. So, many scholars study the expansive soil crack in view of the unsaturated soil theory through the linear elastic mechanics and fracture mechanics. Morris et al. [20] set up the relation with the crack deep, soil character, and the given suction distribution according to different theory. Lee et al. [21], Konrad and Ayad [22], and Zheng et al. [23] established a theoretical model of cohesive soil cracking according to field test and elasticity theory. Sun et al. [24] considered the key factor of cracking is suction because of shrinkage increasing to the tensile strength; they inferred the formula of secondary crack spacing. Li et al. [25] obtained the linear elastic theory relations considering the effective cohesion and effective angle of internal friction.

People study the expansive soil cracking mechanizations using mechanics principle and derivate and establish the calculation model of crack depth, but all of the models are directed against the crack initial depth. Actually, the expansive soil crack increases and deepens under drying and wetting circle, the crack tends to be stable finally. Stability analysis of expansive soil slope involved in crack depth should be based on the crack depth, but no one shows any interest in the crack depth theoretical prediction model under changing environment.

## 6. Future Research Direction

### 6.1. Developing the Means of Observing Crack

Because of cracks, the strength, seepage, and deformation of expansive soil are different from integrated soil. We must consider the germination and process of crack when studying the strength, seepage, and deformation of expansive soil. But we mostly lead the crack into calculation model randomly or by assumption at present. So, the hot spot of expansive soil study in test will be the following: observation and measurement of the crack with the process of the germination study of the influence mechanization of strength, seepage and deformation of expansive soil, quantization of the crack, and so on.

In view of the method of crack measure, we can do it from the following aspects.

1. Resistivity method: test the resistance when current through the soil, the test data can reflect the structure of the soil indirectly; resistivity is an effective means to monitor the crack dynamic development in a certain range. Through measuring the crack at different water content and void ratio many times, we can grasp the influence rule about the water content and void ratio; quantifying and monitoring the crack dynamic development of lossless in the drying and wetting cycles test, we can track the final depth of cracks.
2. Ultrasonic method: measure the ultrasound velocity in the soil by using an ultrasonic pulse transmission method; the different ultrasonic will reflect the interior soil development condition. If we defined the damage variable we can consider the result of macroscopic mechanics through the measuring data of crack depth.
3. CT method: this method is similar to ultrasonic; the two methods can measure the development and germination of cracks dynamic, quantitative and lossless and the measurement of precision is enough to test demand. Instrument of CT is expensive and the operating demand is above the average; the development in expansive soil crack is restricted, so we study the crack only in a few cases. How to spread the technology of CT to test and practice engineering is the key question at present.
4. Liquid flow method: develop a chemistry reagent with high velocity and the reagent can condense quickly starting from the view of hydrodynamics, pour the reagent into cracks after the crack stabilization, and take it out when condensed; the condensed reagent will represent cracks overview of expansive soil at different location; the depth of crack can be measured directly. Using this method for measuring cracks causes no harm to the soil; we also can measure it many times according to requirement.
5. Theoretical calculation method: the crack depth is a dynamic process with drying and wetting cycles, so we want to obtain that the accurate depth of crack is difficult and the deviation is large. Through the theoretical analysis, try to find the calculated method of crack depth when water content, dry density, and cycles index change; we can calculate the crack depth from the obtained surface crack width.
6. Optical image analysis method: through the image acquisition system, including long distance microscope, three axis displacement platforms, CCD camera, and video monitor, we can observe and collect the image of the development process of expansive soil crack, then we can obtain the crack character about the non-contact, continuous, and systemic micro-structural changes. This method has wide range of applications and can be lead into practice engineering; we must avoid jamming as far as possible when managing the collecting image and ensure that the image can react to the development of crack objectively.

### 6.2. Cracks Statistics and Description Methods

Expansive soil engineering mechanics are influenced by geometric elements, such as the trend, dip angle, width, length, and step of crack. Describing and quantifying the crack are based on the basis of existing cracks at present; only current crack morphology of expansive soil can be reflected; there are no relevant reports about the quantitative description method of crack occurrence and development currently. Therefore, we should pay attention to strengthen the quantitative description method of crack occurrence and development, for laying the foundations about implanting crack factors into expansive soil constitutive model.

Because the basic theory of study expansive soil from unsaturated soil is not mature, the test method about measure crack and dynamic characterization is not perfect; there are many deficiencies about the study of the influence of expansive soil character. With the ceaseless development of unsaturated soil mechanics, test instrument, and observation method, cracks quantification and its effect on strength, deformation, permeability and stability of expansive soil, and analysis of quantitative research will be important directions in the future. In addition, it is very necessary that establish cracks occurrence and development of mathematical and physical model through the relevant mechanics and geotechnical tests. Relevant results will improve the basic theory of unsaturated soil mechanics and promote the development of unsaturated soil mechanics.

Amarasiri et al. [26] used the universal distinct element method (UDEM) to simulate the soil crack firstly; the software of UDEM was widely applied in primary analysis; the software along with the Fast Lagrangian Analysis of Continua (FLAC) is perfectly compared with FLAC the superiority. Using UDEM to analyze soil shrinkage cracks will be the main research methods. Moreover, Monte Carlo method (also can say random simulation method) can solve various problems (object of study involves random processes and outcomes); it was used in many respects, such as particle transport, statistics physics, reliability analysis, and military affairs. The development of soil shrinkage crack is a random process; we think that simulating the crack using the Monte Carlo method is effective. At present, Monte Carlo method is used in simulating rock fracture frequently but used in soil crack no report [27–29]. We can try to use the method to study the soil shrinkage cracks in the future.

Existing methods of expansive soil crack observation and representation have certain confines and under the influence of human factor, study on the expansive soil considering crack mostly stays in the qualitative analysis, quantization studies on the crack depth and width are few. The form, distribution, and growth of crack in reality engineering are different from the laboratory test result or hypothesis; in addition, finite element software is short of the quantization model; the model must be considered on the expansive soil crack strength, deformation, permeability, and stability analysis, so the calculation result is different from reality observation. Therefore, it is very necessary to improve the observation method of expansive soil, determine crack quantitative indicators, and strengthen development of software, as soon as we apply the research results in practical engineering widely.

## 7. Conclusion

Expansive soil crack character is the key of slope stability. A number of achievements of crack character are obtained at present, but the conclusions are not agreed with widely. The crack is close together related to seepage, seepage impact on the expansive soil strength and deformation. Hence, improve the method of obtaining crack character image and information processing, carry out crack depth measurement and statistical analysis, study the relationship between depth and surface crack characteristics, and discuss the feedback mechanism of dynamic cracks description, swelling and shrinkage, and overconsolidated characteristics; establish the contact about the process of crack with the expansive soil strength, deformation, penetration and swelling shrinkage. Establishing the expansive soil constitutive model of considering cracks from the point of cracks affects the strength and deformation will be the hot spot of expansive soil study.
